# Decision‐Making Readiness and Its Influencing Factors Among Lung Cancer Patients Receiving Chemotherapy: A Cross‐Sectional Study

**DOI:** 10.1002/nop2.70666

**Published:** 2026-06-29

**Authors:** Yan Wang, Jinping Li, Yumeng Quan, Minfeng Zhai, Xuenan Hu, Panrong Wang, Jia Zhong, Jie Wang, Xin Sun

**Affiliations:** ^1^ Department of Medical Oncology National Cancer Center/National Clinical Research Center for Cancer/Cancer Hospital, Chinese Academy of Medical Sciences and Peking Union Medical College Beijing China; ^2^ School of Nursing Chinese Academy of Medical Sciences & Peking Union Medical College Beijing China; ^3^ Medical Affairs Office National Cancer Center/National Clinical Research Center for Cancer/Cancer Hospital, Chinese Academy of Medical Sciences and Peking Union Medical College Beijing China

**Keywords:** common sense model, decisional conflict, decision‐making participation attitude, decision‐making readiness, influencing factors, lung cancer

## Abstract

**Background:**

Decision‐making readiness is essential for effective patient participation in treatment decisions, especially for lung cancer patients receiving chemotherapy who face repeated complicated treatment choices. However, existing research investigating their level of decision‐making readiness and its associated influencing factors remains inadequate.

**Aims:**

This study aimed to investigate the level of decision‐making readiness among hospitalized lung cancer patients undergoing chemotherapy, and further explore the influencing factors of their decision‐making readiness.

**Materials and Methods:**

A cross‐sectional observational design was adopted. A total of 452 hospitalized lung cancer patients receiving chemotherapy were recruited using convenience sampling. Data were collected via multiple questionnaires: the Chinese Version of the Preparation for Decision Making Scale, Decisional Conflict Scale, Patient Attitude Toward Treatment Decision‐Making Scale, Decision Regret Scale, and a self‐designed questionnaire covering sociodemographic and clinical characteristics. Descriptive statistics, correlation analysis, and hierarchical multiple linear regression were applied for data analysis.

**Results:**

The median score of participants’ decision‐making readiness was 62.00 (56.00, 70.00), representing a moderate overall readiness level. Correlation analysis demonstrated that decision‐making readiness was positively associated with patients’ willingness to participate in treatment decision‐making and negatively associated with decisional conflict. Hierarchical regression revealed that demographic variables could explain 9.2% of the total variance of decision‐making readiness, and the inclusion of decisional conflict and decision participation attitude raised the explanatory power to 31.1%. Economic status, residential location, decisional conflict and participation attitude were identified as significant predictive factors of decision‐making readiness.

**Discussion:**

Lung cancer patients undergoing chemotherapy only possess moderate decision‐making readiness. Compared with demographic characteristics, psychosocial factors (decisional conflict and decision participation attitude) exert a stronger impact on patients’ decision‐making readiness. These findings also support the applicability of the Common Sense Model of Self‐Regulation in the field of cancer treatment decision‐making research.

**Conclusion:**

Targeted nursing decision‐support interventions are urgently needed to assess and elevate lung cancer patients’ decision‐making readiness, which can further promote the practice of patient‐centered treatment decision‐making.

## 1 Introduction

Lung cancer is the most commonly diagnosed malignancy worldwide, with China reporting over 1 million new cases and 740,000 deaths in 2022, making it the leading cause of cancer‐related morbidity and mortality in the country (Bray et al. 2022; Han et al. 2024). As a cornerstone of lung cancer management, chemotherapy is widely applied not only in advanced or metastatic stages but also as neoadjuvant and adjuvant therapy (Ettinger et al. 2019). Its broad application across different disease stages necessitates continuous evaluation and adjustment of treatment strategies. Consequently, treatment decision‐making is a dynamic process that recurs throughout the disease trajectory rather than being confined to a single clinical time point (Sun et al. 2019).

While clinical guidelines provide a foundational framework for these decisions, they must be tailored to accommodate the unique circumstances of individual patients. Beyond guideline‐based clinical considerations, patients are often confronted with complex personal concerns, including treatment‐related side effects, functional impairments, psychological distress, and financial burden, all of which may influence their willingness and readiness to initiate, continue, or modify chemotherapy (Ankolekar et al. 2022). Research indicates that lung cancer patients may experience greater decisional complexity than patients with other cancer types, driven by rapid disease progression, high emotional distress at diagnosis, and limited time to process information (Sullivan et al. 2023; Stacey et al. 2024). This discrepancy between standardized clinical guidance and patients' real‐world decision‐making challenges underscores the importance of a patient‐centred approach to treatment planning that integrates clinical evidence with patients' physical, psychological, socioeconomic and personal contexts.

Despite the well‐recognized value of this patient‐centred paradigm, translating it into routine clinical practice remains challenging. A traditionally paternalistic approach in thoracic oncology may further impede shared decision‐making (Chinese Hospital Association 2019), which in turn contributes to inadequate decision preparation among patients. Such inadequacy has been linked to higher decisional conflict, lower satisfaction and poor treatment adherence (Noteboom et al. 2021). In response to these challenges, patient involvement in treatment decision‐making has gained increasing recognition in oncology practice as a critical strategy to improve decision quality and patient outcomes. However, effective and meaningful patient involvement hinges on adequate decision‐making readiness (DMR).

DMR refers to the comprehensive preparedness of an individual in terms of knowledge, attitude, ability, and environmental support when facing a decision‐making situation, enabling effective participation in the decision‐making process and informed choices (Kalantar‐Zadeh and Zisman‐Ilani 2025). However, existing studies in oncology research, both domestically and internationally, have mainly focused on decisional conflict, shared decision‐making, and decision outcomes, while less attention has been paid to decision‐making readiness as a relatively independent patient psychological indicator (Su et al. 2025; Dalmia et al. 2022; Heirman et al. 2024; Cooley et al. 2022). In particular, relevant evidence targeting lung cancer patients undergoing chemotherapy remains insufficient, and the theoretical interpretation of DMR is still limited.

To further clarify the psychological mechanism underlying patients' DMR, this study adopts the Common Sense Model of Self‐Regulation (CSM) as the theoretical framework. The CSM explains how individuals form illness perceptions, adopt coping behaviours, and evaluate health outcomes when facing health threats (Cannon et al. 2022; Riley et al. 2020; Bennett et al. 2010). This theoretical framework is inherently suitable for interpreting patients' treatment decision‐making: patients first form cognitive and emotional perceptions of their disease and chemotherapy, then adopt corresponding coping behavioural responses, which together determine their level of decision‐making readiness. Therefore, DMR can be reasonably interpreted as a behavioural coping outcome under the CSM theoretical pathway. Based on this theoretical logic, this study focuses on the cognitive and coping stages of CSM. The cognitive stage is reflected in patients' decision‐making attitude, decisional conflict, and decisional regret, while the coping stage is embodied in the overall level of DMR. This theoretical matching helps to construct a clear explanatory framework for individual differences in lung cancer patients' decision‐making readiness.

Overall, there are several obvious research gaps in the current literature. First, international and domestic studies mostly focus on negative decision‐related emotions while neglecting the positive adaptive indicator of decision‐making readiness. Second, most relevant studies remain at the descriptive level, lacking theoretical framework support to explain the formation mechanism of DMR (Pearce et al. 2025). Third, few studies have explored the predictive factors of DMR specifically among lung cancer chemotherapy patients within a classic health theoretical model. Filling these gaps is necessary to enrich the theoretical basis of patient decision‐making research and provide evidence for clinical intervention.

This study aims to assess DMR among patients undergoing chemotherapy for lung cancer and to examine how psychological factors—including decision‐making attitude, decisional conflict, and decisional regret—influence DMR within the CSM framework. We hypothesize that decision‐making attitude, decisional conflict and decision regret are significant predictors of DMR among lung cancer patients.

## 2 Methods

### 2.1 Design, Sample and Setting

A cross‐sectional descriptive study was conducted using convenience sampling. Participants were recruited between August and October 2024 from the oncology department of a specialized cancer hospital in Beijing. The inclusion criteria were as follows: (1) pathologically confirmed diagnosis of lung cancer; (2) age ≥ 18 years; (3) ability to understand the questionnaire content, as assessed by a trained clinical nurse; and (4) provision of informed consent with voluntary agreement to participate. The exclusion criteria were: (1) critically ill patients who had discontinued chemotherapy; (2) patients with a documented history of psychiatric disorders or severe cognitive impairment; and (3) patients who were unaware of their diagnosis and whose medical decisions were made entirely by family members.

Sample size calculation was conducted following the rule that no fewer than 10 participants are needed for each predictor variable (Riley et al. 2020). Accordingly, 25 candidate predictors required a minimum of 250 participants. With an anticipated 20% rate of invalid questionnaires, the minimum required sample size was determined to be 313. Ultimately, 452 valid participants were included in this study, which adequately satisfied and surpassed the required sample size standard.

### 2.2 Measures

A structured questionnaire was developed based on findings from previous studies and the clinical characteristics of patients with lung cancer. To ensure content relevance and clarity, expert consultations, a pilot survey, and iterative revisions were conducted. The final questionnaire was tailored to the treatment decision‐making context of lung cancer patients and consisted of the following parts.

#### 2.2.1 Demographic Questionnaire

The sociodemographic information included gender, age, marital status, ethnicity, educational level, primary caregiver, and so forth. Clinical contextual variables included treatment cycle, pathological type, treatment regimen, cancer stage, lines of therapy, history of lung surgery and number of concurrent medications.

#### 2.2.2 Chinese Version of the Preparation for Decision Making Scale (C‐Prep DM)

The C‐Prep DM was originally developed by Canadian nursing scholars and subsequently revised by Bennett et al. (Bennett et al. 2010). The present study utilized its Chinese version, which was translated and psychometrically validated by Li Yu (Li 2017). This scale was applied to measure decision‐making readiness (DMR), aiming to evaluate patients' readiness for health‐related decision‐making and the effectiveness of decision support interventions. It contains 10 items rated on a 5‐point Likert scale, with scores ranging from 1 (‘not at all’) to 5 (‘a great deal’). The total scale score is obtained by multiplying the summed item scores by 4, resulting in a total score range of 20 to 100. Higher scores indicate a higher level of decision‐making readiness. The Cronbach's *α* coefficient of the Chinese version was 0.946 (Li 2017). In the current study, the Cronbach's *α* value of this scale was 0.903.

#### 2.2.3 Patient Attitude Toward Treatment Decision‐Making Scale

This scale was originally developed by Finnish scholar Sainio et al., and its Chinese version was translated and psychometrically validated by Ma Lili in 2004 (Ma 2004). The validated Chinese version was adopted in this study to measure decision‐making attitude (DMA). It consists of 12 items scored on a 3‐point scale (very important = 3, somewhat important = 2, not important = 1). Higher scores indicate a more positive decision‐making attitude toward participation in treatment decisions. The Cronbach's *α* coefficient of the Chinese version was 0.8506 (Ma 2004). In the current study, the Cronbach's *α* value of this scale was 0.837.

#### 2.2.4 Decisional Conflict Scale (DCS)

This scale was initially constructed by O'Connor et al., with its Chinese version translated and validated by Li Yu (Li 2017). The validated Chinese scale consists of 16 items grouped into three dimensions, namely information and values clarity, decision support and effectiveness, and decisional uncertainty. Items are rated on a 5‐point Likert scale and converted to a total score from 0 to 100. Higher scores indicate greater decisional conflict, with scores above 25 indicating the presence of conflict, and scores above 37.5 suggesting possible decision delay. The scale's Cronbach's α coefficient was 0.897 (Li 2017). In the current study, the Cronbach's α value of this scale was 0.938.

#### 2.2.5 Decision Regret Scale (DRS)

This scale was formulated by Brehaut et al. (Brehaut et al. 2003), and its standardized Chinese version was completed and verified by Chen et al. (Chen and Cheng 2018). The present study adopted this validated Chinese scale, which contains 5 items scored via a 5‐point Likert scale from 1 (strongly agree) to 5 (strongly disagree). Among all items, item 2 and item 4 adopt reverse scoring. The total score is calculated using the formula: (mean score of all items—1) × 5, yielding a range from 0 to 20. Higher scores indicate greater regret related to the treatment decision. The Cronbach's *α* coefficient was 0.826 (Chen and Cheng 2018). In the current sample, the Cronbach's *α* coefficient of the Decision Regret Scale was 0.401, indicating relatively low internal consistency. Therefore, all statistics results and conclusions related to DRS should be interpreted with appropriate caution. Nevertheless, considering its brief item structure and clear theoretical connotation, this variable was still retained for exploratory correlation analysis to enrich the research framework and supplement theoretical discussion, which was consistent with our preset research design.

### 2.3 Data Collection

Data were collected using a structured electronic questionnaire. Prior to data collection, the principal investigator provided standardized training to designated nurses responsible for recruitment and questionnaire administration, ensuring consistency in study explanations and instructions. Trained research nurses screened newly admitted and currently hospitalized lung cancer patients receiving chemotherapy on a daily basis. Eligible patients were informed about the study purpose, procedures, confidentiality protection, and voluntary participation before written informed consent was obtained.

To ensure data quality, each electronic device was permitted to submit the questionnaire only once. Clear and standardized instructions were provided for each section of the questionnaire, and the average completion time was approximately 15 min. If participants encountered any difficulties during completion, trained nurses provided assistance without influencing participants' responses. These procedures helped ensure the standardization, completeness, and reliability of the collected data.

### 2.4 Statistical Analysis

Statistical analyses were conducted using SPSS version 26.0 (IBM Corp., Armonk, NY, USA). Continuous variables were tested for normality using the Shapiro–Wilk test. Normal distribution was expressed as mean ± standard deviation (SD), while non‐normally distributed data were presented as median and interquartile range (*M*[P25, P75]). For comparisons between two groups, independent samples *t*‐tests were used for normally distributed data, and the Wilcoxon rank‐sum test was applied for non‐normally distributed data. For comparisons among three or more groups, one‐way analysis of variance (ANOVA) was used for normally distributed data, and the Kruskal–Wallis test was used for non‐normally distributed data. Correlations between variables were examined using Pearson correlation for continuous normally distributed variables and Spearman correlation (*r*_s_) for ordinal or non‐normally distributed variables.

Collinearity diagnostics were conducted for DMA, DCS and DRS, and the corresponding VIF values ranged from 1.197 to 1.409, confirming the absence of severe multicollinearity among these related psychological variables. Complete regression diagnostic results and relevant plots were provided in the Supporting Information.

Hierarchical multiple linear regression analysis guided by the Common Sense Model of Self‐Regulation was conducted to explore factors associated with decision‐making readiness (DMR). Variables were incorporated following a theory‐based entry sequence and preliminary univariate analysis outcomes. Sociodemographic and clinical contextual variables were placed in the first block to adjust for background factors affecting patients' decision‐making conditions and accessible resources, while decision‐related psychological variables including decision‐making attitude and decisional conflict were added into the second block to explore their extra explanatory power for DMR beyond contextual factors. All statistical tests were two‐tailed, and a *p* < 0.05 was considered statistically significant. Decision regret was excluded from the final regression model, as it is defined as a post‐decision evaluation indicator and showed no significant correlation with DMR in this sample.

Missing values were processed as follows: in statistical analyses. For any scale with less than 20% missing items, missing data were imputed using each participant's mean score of the remaining valid items. Questionnaires with 20% or more missing items on a given scale were excluded from further analysis. In this study, 9 questionnaires were removed due to incomplete and invalid responses, leaving a final sample of 452 participants.

## 3 Results

### 3.1 General Information and Univariate Analysis of DMR Among Lung Cancer Patients During Chemotherapy

A total of 461 questionnaires were collected, of which 452 were complete and valid, yielding an effective response rate of 98.05%. Among the participants, 325 (71.9%) were male and 127 (28.1%) were female. A total of 161 patients (35.6%) were younger than 60 years, and 291 (64.4%) were aged 60 or older. Regarding marital status, 5 (1.1%) were unmarried, 365 (80.8%) were married, and 82 (18.1%) were divorced or widowed. Detailed demographic and clinical characteristics were presented in Table 1. Univariate analysis revealed statistically significant differences in DMR across age groups, residence, and number of concurrent medications used (*p* < 0.05) (Table 1).

**TABLE 1:** General information and univariate analysis of DMR among lung cancer patients during chemotherapy (*n* = 452).

Variables	Categories	*n* (%)	DMR (*M*[P25, P75])	Statistic	*p*
Total		452 (100)	62.00 (56.00, 70.00)		
Gender	Men	325 (71.90)	62.00 (56.00, 70.00)	*Z* = −0.00	0.997
	Women	127 (28.10)	62.00 (56.00, 72.00)		
Age (years)	18–59	161 (35.62)	64.00 (58.00, 72.00)	*Z* = −2.24	0.025
	≥ 60	291 (64.38)	62.00 (56.00, 70.00)		
Marital status	Unmarried	5 (1.11)	62.00 (58.00, 84.00)	*χ* ^2^ = 1.32^#^	0.516
	Married	365 (80.75)	62.00 (56.00, 72.00)		
	Divorce/widow	82 (18.14)	62.00 (56.00, 68.00)		
Ethnicity	Han ethnicity	420 (92.92)	62.00 (56.00, 70.00)	*Z* = −0.52	0.600
	Ethnic minorities	32 (7.08)	64.00 (56.00, 73.00)		
Education level	Primary school	53 (11.73)	62.00 (56.00, 68.00)	*χ* ^2^ = 3.55^#^	0.170
	Secondary school/vocational school	260 (57.52)	64.00 (56.00, 74.00)		
	College degree or above	139 (30.75)	62.00 (56.00, 70.00)		
Primary caregiver	Spouse	267 (59.07)	62.00 (56.00, 71.00)	*χ* ^2^ = 0.50^#^	0.778
	Children	116 (25.66)	62.00 (58.00, 70.50)		
	Alone/others	69 (15.27)	62.00 (56.00, 70.00)		
Economic status	< 4000	176 (38.94)	62.00 (56.00, 68.00)	*χ* ^2^ = 5.75^#^	0.056
	4000–6000	176 (38.94)	62.00 (56.00, 70.50)		
	> 6000	100 (22.12)	64.00 (58.00, 76.00)		
Occupation	Retired	147 (32.52)	62.00 (54.00, 70.00)	*χ* ^2^ = 4.84^#^	0.184
	Employees of enterprises/institutions	98 (21.68)	64.00 (58.00, 71.50)		
	Farmer	97 (21.46)	62.00 (56.00, 70.00)		
	Unemployed/self‐employed	110 (24.34)	64.00 (58.00, 71.50)		
Residence	Urban	251 (55.53)	62.00 (56.00, 68.00)	*χ* ^2^ = 8.65^#^	0.013
	Country side	42 (9.29)	65.00 (58.00, 78.50)		
	Town	159 (35.18)	64.00 (58.00, 74.00)		
Medical insurance	New Rural Cooperative Medical Scheme	112 (24.78)	62.00 (56.00, 70.00)	*χ* ^2^ = 2.72^#^	0.257
	Urban Employee/Resident Basic Medical Insurance	320 (70.80)	62.00 (56.00, 70.50)		
	Others	20 (4.42)	66.00 (60.00, 73.00)		
Treatment cycles	First cycle	66 (14.60)	62.00 (56.50, 67.50)	*χ* ^2^ = 1.37^#^	0.505
	2–4 cycles	166 (36.73)	64.00 (56.50, 72.00)		
	5 or more cycles	220 (48.67)	62.00 (56.00, 70.00)		
Pathological type	Non‐small cell lung cancer	293 (64.82)	62.00 (56.00, 72.00)	*Z* = −0.75	0.453
	Small cell lung cancer	159 (35.18)	62.00 (56.00, 68.00)		
Treatment regimen	Monotherapy	123 (27.21)	62.00 (56.00, 70.00)	*Z* = −0.38	0.707
	Combination therapy	329 (72.79)	62.00 (56.00, 72.00)		
Cancer stage	I	23 (5.09)	64.00 (60.00, 70.00)	*χ* ^2^ = 1.44^#^	0.695
	II	32 (7.08)	64.00 (58.00, 68.50)		
	III	124 (27.43)	62.00 (58.00, 72.00)		
	IV	273 (60.40)	62.00 (56.00, 70.00)		
Lines of therapy	First‐line	307 (67.92)	62.00 (56.00, 70.00)	*χ* ^2^ = 1.06^#^	0.589
	Second to third‐line	118 (26.11)	62.00 (56.00, 72.00)		
	Multi‐line	27 (5.97)	60.00 (53.00, 67.00)		
History of lung surgery	No	292 (64.60)	62.00 (56.00, 72.00)	*Z* = −1.08	0.279
	Yes	160 (35.40)	62.00 (55.50, 70.00)		
Number of concurrent medications	None	61 (13.50)	64.00 (58.00, 80.00)	*χ* ^2^ = 11.37^#^	0.003
	1–2 types	273 (60.40)	62.00 (54.00, 68.00)		
	≥ 3 types	118 (26.11)	64.00 (58.50, 70.00)		

Abbreviations: *M*, median; Q_1_, 1st Quartile; Q_3_, 3rd Quartile.

^#^ Kruskal–Waills test.

### 3.2 Scores of Scales Among Lung Cancer Patients During Chemotherapy

The median total score of the DMR among 452 lung cancer patients undergoing chemotherapy was 62.00 (56.00, 70.00), indicating a moderate level of preparedness. The median scores for the other scales were as follows: DMA 29.00 (26.00, 32.00), DCS 32.00 (23.25, 36.00) and DRS 9.00 (8.00, 11.00). Descriptive statistics for all scales are summarized in Table 2.

**TABLE 2:** Scores of scales for lung cancer patients during chemotherapy.

Dimension	Number of items	Score range	Total score	Average item score
DMR	10	20–100	62.00 (56.00, 70.00)	6.20 (5.60, 7.00)
DMA	12	12–36	29.00 (26.00, 32.00)	5.90 (4.86, 6.94)
DCS	16	0–100	32.00 (23.25, 36.00)	2.00 (1.45, 2.25)
DRS	5	0–20	9.00 (8.00, 11.00)	1.80 (1.60, 2.20)

### 3.3 Correlation Analysis of DMR Among Lung Cancer Patients During Chemotherapy

Spearman correlation analysis demonstrated that DMR was positively correlated with DMA (*r* = 0.382, *p* < 0.001), and negatively correlated with the total score and all subscales of the DCS (*r* = −0.212 to −0.164, *p* < 0.001). The detailed results were shown in Table 3.

**TABLE 3:** Correlation between DMR and DCS, DMA and DRS in lung cancer patients during chemotherapy.

Dimension	Total score of DMA	Information and values clarity	Decision support and effectiveness	Decisional uncertainty	Total score of DCS	Total score of DRS
Total score of DMR	0.382**	−0.197**	−0.212**	−0.185**	−0.164**	0.005

*Note:* All values are Spearman's rank correlation coefficients (*r*_s_).

** *p* < 0.001.

### 3.4 Multivariate Analysis of DMR Among Lung Cancer Patients During Chemotherapy

The total DMR score was treated as the dependent variable. Independent variables included factors identified as statistically significant in univariate and correlation analyses, as well as clinically contextual variables supported by existing literature, even if they were not statistically significant in univariate analysis (*p* > 0.05). A hierarchical multiple linear regression analysis was performed.

Prior to formal regression analysis, we examined all assumptions for the regression model. The Durbin‐Watson statistic was 1.987, indicating independence of residuals. All cases had a Cook's distance below 1, suggesting no influential observations. Although the Shapiro–Wilk test reached statistical significance (*p* < 0.05) due to the large sample size, the points on the residual Q‐Q plot were roughly distributed along the diagonal line, which supported the approximate normality of residuals. The scatter plot of standardized residuals against predicted values showed that residuals were evenly distributed around zero with no obvious heteroscedasticity. Collinearity diagnostics revealed that the variance inflation factor (VIF) of all independent variables was less than 2, indicating no serious multicollinearity. Complete regression diagnostic results and relevant plots were provided in the Supporting Information.

Dummy variables were created for nominal categorical variables, and the coding scheme was presented in Table 4.

**TABLE 4:** Variable assignments of factors.

Variable	Coding method
Age (years)	18–59 = 1, ≥ 60 = 2
Economic status	Dummy variables were created using ‘< 4000 RMB’ as the reference group
Residence	Dummy variables were created using ‘urban’ as the reference group
Number of concurrent medications	Dummy variables were created using ‘none’ as the reference group
DCS	Entered as a continuous variable
DMA	Entered as a continuous variable

In the hierarchical regression model, demographic characteristics and number of concurrent medications were entered in the first block and accounted for 9.2% of the variance in DMR (*R* ^2^ = 0.092, *p* < 0.001). In the second block, DCS and DMA were added, increasing the explained variance to 31.1% (*R* ^2^ = 0.311, ∆*R* ^2^ = 0.219, *p* < 0.001). The final model demonstrated that economic status, place of residence, decisional conflict, and decision‐making attitude were significant predictors of DMR among lung cancer patients undergoing chemotherapy (*p* < 0.05), as detailed in Table 5.

**TABLE 5:** Hierarchical analysis of DMR among lung cancer patients during chemotherapy (*n* = 452).

Model	Variable	*B*	SE	*β*	*t*	*p*	95% CI
1	Constant	32.618	1.099		29.676	< 0.001	30.458 to 34.778
	Age	−1.117	0.624	−0.081	−1.792	0.074	−2.343 to 0.108
	Residence (Ref = Urban)						
	Country side	4.046	1.132	0.178	3.575	< 0.001	1.822 to 6.27
	Town	2.531	0.69	0.184	3.671	< 0.001	1.176 to 3.887
	Economic status (Ref ≤ 4000 RMB)						
	4000–6000 RMB	2.034	0.717	0.151	2.838	0.005	0.625 to 3.442
	> 6000 RMB	3.387	0.864	0.214	3.921	< 0.001	1.689 to 5.085
	Number of concurrent medications (Ref = None)						
	1–2 types	−3.056	0.903	−0.227	−3.384	0.001	−4.830 to −1.281
	≥ 3 types	−2.365	1.003	−0.158	−2.359	0.019	−4.335 to −0.395
2	Constant	24.739	2.623		9.432	< 0.001	19.585 to 29.894
	Age	−0.727	0.548	−0.053	−1.325	0.186	−1.804 to 0.351
	Residence (Ref = Urban)						
	Country side	3.119	0.991	0.138	3.147	0.002	1.172 to 5.067
	Town	2.193	0.603	0.159	3.636	< 0.001	1.008 to 3.378
	Economic status (Ref ≤ 4000 RMB)						
	4000–6000 RMB	1.552	0.627	0.115	2.474	0.014	0.319 to 2.784
	> 6000 RMB	1.766	0.768	0.111	2.300	0.022	0.257 to 3.274
	Number of concurrent medications (Ref = None)						
	1–2 types	−0.299	0.822	−0.022	−0.364	0.716	−1.914 to 1.316
	≥ 3 types	0.817	0.921	0.054	0.887	0.376	−0.994 to 2.628
	DMA	0.390	0.069	0.250	5.672	< 0.001	0.255 to 0.526
	DCS	−0.193	0.025	−0.347	−7.618	< 0.001	−0.242 to −0.143

*Note:* In the first step, *R* ^2^ = 0.092, *p* < 0.001; in the second step, *R* ^2^ = 0.311, Δ*R* ^2^ = 0.219, *p* < 0.001.

## 4 Discussion

### 4.1 Status of DMR Among Lung Cancer Patients During Chemotherapy

This study found that lung cancer patients during chemotherapy had a moderate level of decision‐making readiness, with a median score of 62.00 (56.00, 70.00). This is consistent with previous findings reported by Zhang et al. (Zhang et al. 2024), but lower than the scores observed by Tan et al., who studied young and middle‐aged female cancer patients (Tan et al. 2023). According to the CSM, older patients may have lower illness perception or reduced confidence in treatment benefits, which along with differences in health literacy and treatment decision behaviours, these factors are associated with the lower DMR observed in this study (Huang et al. 2023). In addition, methodological differences—such as the use of different scales with PrepDM in Tan et al. Emphasizing communication and value weighting versus the C‐PrepDM used here focusing on information needs and risk communication—may also explain these variations.

Building on these findings, tailored nursing interventions are essential to enhance patients' DMR for treatment decisions. Evidence suggests that nurse‐led engagement interventions—ranging from generic and patient‐specific information delivery to personalized decision support and motivational support—can significantly improve patient activation, informed decision‐making, health literacy, and quality of life among adults with cancer (Bonetti et al. 2022). Within this framework, structured educational tools, such as decision aids, brochures, and videos, may further enhance patients' understanding of treatment options and associated risks and benefits, facilitating deliberation, preference clarification, and active participation in decision‐making. Routine assessment of DMR throughout the chemotherapy process, coupled with individualized education and decision support, is recommended to strengthen patient confidence and participation in care planning.

### 4.2 Multilevel Determinants of Decision‐Making Readiness Within the CSM Framework

#### 4.2.1 Socioeconomic Context Shapes Cognitive and Coping Resources for DMR

The study found that economic status and place of residence were significantly associated with DMR among lung cancer patients during chemotherapy. Patients with a monthly income of ≥ 4000 RMB had significantly higher DMR (*p* < 0.05), consistent with previous findings (Lu 2022). From the perspective of the social determinants of health, socioeconomic resources influence not only access to healthcare services but also patients' capacity to process information and engage in decision‐making (Song et al. 2025; Liu 2022).

Within the CSM framework, economic advantage correlates with stronger patients' cognitive representations of illness, higher perceived controllability and treatment efficacy, as well as more adaptive coping responses and higher DMR (Hagger and Orbell 2022; Mai et al. 2023). Patients with greater financial resources may experience less treatment‐related uncertainty and anxiety, allowing them to allocate more cognitive and emotional resources to deliberation and active participation in decision‐making.

Notably, patients residing in rural or township areas exhibited higher DMR than those in urban settings. This finding contrasts with assumptions that urban patients necessarily demonstrate greater autonomy in medical decision‐making. In a collectivist cultural context such as China, rural patients may benefit from stronger family involvement and shared responsibility in healthcare decisions, which can reduce decisional burden and enhance perceived support. Within the CSM, such social support is correlated with lower emotional distress and improved coping strategies, as well as greater readiness to engage in treatment decisions.

#### 4.2.2 Decision‐Making Participation Attitude as a Cognitive‐Motivational Correlate of DMR

A positive association was observed between DMA and DMR, indicating that patients who value involvement in treatment decisions are more prepared to engage in decision‐making processes. This finding aligns with previous studies suggesting that positive decisional attitudes are linked to active participation in care (Ma 2004).

From the perspective of CSM, DMA reflects patients' cognitive appraisal of their role in illness management and their perceived self‐efficacy in influencing treatment outcomes. When patients perceive participation as meaningful and within their capability, they are more likely to adopt active coping strategies, which correlate with higher readiness for decision‐making.

These findings suggested that DMA is not merely an individual preference but a modifiable cognitive‐motivational construct. Interventions aimed at enhancing patients' role clarity, confidence, and perceived legitimacy in decision‐making may therefore strengthen DMR, particularly among patients who initially adopt a passive stance. Future intervention studies are warranted to test whether targeted strategies aimed at enhancing decision‐making participation attitudes could improve DMR and related patient outcomes.

#### 4.2.3 Decisional Conflict as a Dynamic Correlate of DMR

Decisional conflict was negatively associated with DMR, suggesting that patients experiencing greater uncertainty and value ambiguity were less prepared to engage in treatment decisions. This finding is consistent with previous research demonstrating that decisional conflict is linked to poorer patient engagement and satisfaction. Share decision‐making processes have been shown to reduce decisional conflict and improve decision outcomes in oncology contexts (Sui 2021; Tan et al. 2022).

Within the framework of CSM, decisional conflict correlates with inadequate cognitive representation and compromised coping capacity. Existing studies across other health populations have also found higher decisional conflict to be associated with greater illness uncertainty and lower disease knowledge (Sabin et al. 2024). A high level of conflict reflects unresolved discrepancies between perceived risks, benefits, and personal values, which are associated with barriers to the transition from cognitive appraisal to action‐oriented coping. Conversely, higher DMR is correlated with better information integration and value clarification, as indicated by longitudinal research on cancer patients' decisional conflict trajectories (Wu et al. 2022).

Although causality cannot be inferred from this cross‐sectional design, these findings support the conceptualization of decisional conflict and DMR as dynamically interrelated processes. Longitudinal studies are warranted to examine their reciprocal influence across the treatment trajectory.

### 4.3 Integrating Dynamic, Multilevel Implications for DMR

Overall, these findings indicate that DMR is a dynamic, modifiable construct rather than a static trait. Patients' cognitive representations, coping strategies, and perceived self‐efficacy evolve throughout the chemotherapy trajectory, and DMR varies alongside disease progression, treatment experiences, and resource availability. For example, patients may initially have low DMR due to limited information or anxiety, but repeated education, symptom management, and supportive interactions can enhance cognitive appraisal and coping, which are associated with enhanced readiness. Conversely, adverse events, changes in family support, or new health information may temporarily reduce DMR, underscoring this dynamism.

Socioeconomic context, DMA, and decisional conflict appear to interact and jointly correlate with DMR, and collectively relate to how patients perceive their role, process information, and engage in decision‐making. This integrative perspective identifies potential targets for tailored interventions, including structured decision aids, literacy‐sensitive educational materials, value clarification exercises, and family‐inclusive counselling. Repeated assessment of DMR across chemotherapy cycles can help clinicians identify periods of lower readiness and provide timely, individualized support, ultimately reducing decisional conflict and promoting active patient participation.

Future longitudinal and interventional studies are warranted to examine how DMR evolves over the treatment trajectory, to validate the effectiveness of tailored interventions, and to explore potential mediators and moderators, such as DMA and decisional conflict across diverse cultural and socioeconomic contexts. By emphasizing the dynamic and multilevel nature of DMR, such research can guide the development of more effective patient‐centered strategies in oncology care.

### 4.4 Practice Implications

DMR should be regarded as a key component of nursing assessment for lung cancer patients undergoing chemotherapy. Given its close association with decisional conflict and decision‐making participation attitude, medical staff should focus on identifying patients with low readiness and providing timely, targeted decision support.

Nursing strategies that emphasize clear information delivery, value clarification, and family involvement may help reduce decisional conflict and strengthen patients' engagement in treatment decision‐making, thereby supporting patient‐centred care.

## 5 Limitation and Future Directions

This study has several limitations. First, its cross‐sectional design limits causal inference between decision‐making readiness and related psychological factors. Longitudinal studies are needed to examine change in DMR across the chemotherapy trajectory. Second, participants were recruited from a single oncology hospital using convenience sampling, which may limit generalizability. Multicentre studies with more diverse samples are recommended. Third, reliance on self‐reported measures may introduce response bias. Future research could incorporate qualitative or mixed‐method approaches to capture the decision‐making process more comprehensively. Fourth, the Decision Regret Scale exhibited low internal consistency in the present sample. Relevant analytical results should therefore be interpreted with caution, and further validation of this scale is suggested in future studies. Finally, intervention studies are warranted to test nurse‐led strategies targeting modifiable factors such as decisional conflict and decision‐making participation attitude to enhance DMR and patient‐centred outcomes.

## 6 Conclusion

In summary, lung cancer patients undergoing chemotherapy exhibit a moderate, dynamic level of decision‐making readiness. DMR is associated with psychological factors such as decision‐making attitude and decisional conflict, as well as demographic factors including economic status and place of residence. Psychological influences appear particularly prominent, supporting the applicability of the CSM in this context. These findings underscore the need for tailored nursing interventions that address cognitive, emotional, and social dimensions to enhance readiness, facilitate patient‐centered treatment decisions, and ultimately improve adherence and clinical outcomes.

## Author Contributions

All authors contributed to the study conception and design. Conceptualization: Yan Wang and Jinping Li. Data curation: Yan Wang, Xin Sun, Jinping Li and Yumeng Quan. Formal analysis: Jinping Li and Yumeng Quan. Investigation: Jinping Li, Minfeng Zhai, Xuenan Hu and Panrong Wang. Methodology: Xin Sun, Yan Wang, Jinping Li, Yumeng Quan and Panrong Wang. Visualization: Xin Sun, Yan Wang and Jia Zhong. Writing – original draft: Yan Wang. Writing – review and editing: Yan Wang, Jinping Li and Yumeng Quan. Funding acquisition: Yan Wang and Jie Wang. Project administration: Yan Wang and Xin Sun. Supervision: Xin Sun.

## Funding

This work was supported by Noncommunicable Chronic Diseases‐National Science and Technology Major Project (2024ZD0519700/2024ZD0519704 to Jie Wang); NSFC General Program Online (82272796 to Jie Wang); Beijing Municipal Science and Technology Program (Z231100004823018 to Jie Wang); Y&M Cultivation Project of Cancer Hospital Chinese Academy of Medical Sciences (Grant/Award Number: CICAMS‐MOY&M‐202404).

## Ethics Statement

The study protocol was reviewed and approved by the Ethics Committee of the Cancer Hospital, Chinese Academy of Medical Sciences (Approval No. 24/439‐4719 and Date 2024‐09‐12) in accordance with the ethical standards of the Declaration of Helsinki.

## Consent

Informed consent was obtained from all participants prior to data collection.

## Conflicts of Interest

The authors declare no conflicts of interest.

## Supporting information


**Table S1:** Collinearity diagnostics of the DMA, DCS and DRS.
**Figure S1:** Scatter plot of unstandardized residuals versus unstandardized predicted values.
**Figure S2:** Normal Q–Q Plot of unstandardized residuals for the Hierarchical Regression Model.

**Table S2:** Model summary of the Hierarchical Regression Analysis.
**Figure S3:** Normal Q–Q plot of unstandardized residuals for the Hierarchical Regression Model.
**Figure S4:** Scatter plot of studentized residual versus unstandardized predicted value.
**Table S3:** Coefficients table with collinearity statistics.

## Data Availability

The datasets generated and analysed during the current study are available from the corresponding author upon reasonable request. Due to ethical considerations, the data are not publicly accessible.

## References

[nop270666-bib-0001] Ankolekar, A. , B. van der Heijden , A. Dekker , et al. 2022. “Clinician Perspectives on Clinical Decision Support Systems in Lung Cancer: Implications for Shared Decision‐Making.” Health Expectations 25, no. 4: 1342–1351. 35535474 10.1111/hex.13457 PMC9327823

[nop270666-bib-0002] Bennett, C. , I. D. Graham , E. Kristjansson , S. A. Kearing , K. F. Clay , and A. M. O'Connor . 2010. “Validation of a Preparation for Decision Making Scale.” Patient Education and Counseling 78, no. 1: 130–133. 19560303 10.1016/j.pec.2009.05.012

[nop270666-bib-0003] Bonetti, L. , A. Tolotti , G. Anderson , et al. 2022. “Nursing Interventions to Promote Patient Engagement in Cancer Care: A Systematic Review.” International Journal of Nursing Studies 133: 104289. 10.1016/j.ijnurstu.2022.104289. 35751947

[nop270666-bib-0004] Bray, F. , M. Laversanne , H. Sung , et al. 2022. “Global Cancer Statistics 2022: GLOBOCAN Estimates of Incidence and Mortality Worldwide for 36 Cancers in 185 Countries.” CA: A Cancer Journal for Clinicians 74, no. 3: 229–263. 10.3322/caac.21834 38572751

[nop270666-bib-0005] Brehaut, J. C. , A. M. O'Connor , T. J. Wood , et al. 2003. “Validation of a Decision Regret Scale.” Medical Decision Making 23, no. 4: 281–292. 12926578 10.1177/0272989X03256005

[nop270666-bib-0006] Cannon, M. , M. Credé , J. M. Kimber , A. Brunkow , R. Nelson , and M. A. LM . 2022. “The Common‐Sense Model and Mental Illness Outcomes: A Meta‐Analysis.” Clinical Psychology & Psychotherapy 29, no. 4: 1186–1202. 35112427 10.1002/cpp.2721

[nop270666-bib-0007] Chen, F. , and X. Q. Cheng . 2018. “Reliability and Validity Evaluation of the Chinese Version of the Decisional Regret Scale Applied to Facial Cosmetic Surgery Patients.” Journal of Nursing 25, no. 7: 42–44.

[nop270666-bib-0008] Chinese Hospital Association . 2019. “Patient Safety Goals (2019 Edition).” Chinese Health 12: 57–58.

[nop270666-bib-0009] Cooley, M. E. , E. Mazzola , N. Xiong , et al. 2022. “Clinical Decision Support for Symptom Management in Lung Cancer Patients: A Group RCT.” Journal of Pain and Symptom Management 63, no. 4: 572–580. 34921934 10.1016/j.jpainsymman.2021.12.006 PMC9194912

[nop270666-bib-0010] Dalmia, S. , F. Boele , K. Absolom , et al. 2022. “Shared Decision Making in Early‐Stage Non‐Small Cell Lung Cancer: A Systematic Review.” Annals of Thoracic Surgery 114, no. 2: 581–590. 10.1016/j.athoracsur.2021.01.046. 33581150

[nop270666-bib-0011] Ettinger, D. S. , D. E. Wood , D. L. Aisner , et al. 2019. “Non‐Small Cell Lung Cancer, Version 3.2022, NCCN Clinical Practice Guidelines in Oncology.” Journal of the National Comprehensive Cancer Network 20, no. 5: 497–530. 10.6004/jnccn.2022.0025 35545176

[nop270666-bib-0012] Hagger, M. S. , and S. Orbell . 2022. “The Common Sense Model of Illness Self‐Regulation: A Conceptual Review and Proposed Extended Model.” Health Psychology Review 16, no. 3: 347–377. 33461402 10.1080/17437199.2021.1878050

[nop270666-bib-0013] Han, B. , R. Zheng , H. Zeng , et al. 2024. “Cancer Incidence and Mortality in China, 2022.” Journal of the National Cancer Center 4, no. 1: 47–53. 39036382 10.1016/j.jncc.2024.01.006 PMC11256708

[nop270666-bib-0014] Heirman, A. N. , C. R. Arends , D. V. C. de Jel , et al. 2024. “Decisional Conflict and Decision Regret in Head and Neck Oncology: A Systematic Review and Meta‐Analysis.” JAMA Otolaryngology. Head & Neck Surgery 150, no. 5: 393–404. 38512270 10.1001/jamaoto.2024.0214 PMC10958390

[nop270666-bib-0015] Huang, Y. , Y. Liu , Y. Wang , and M. Huang . 2023. “Research Progress of the Common‐Sense Model of Self‐Regulation in the Management of Chronic Diseases.” Chinese Journal of Nursing 58, no. 18: 2293–2298.

[nop270666-bib-0016] Kalantar‐Zadeh, K. , and Y. Zisman‐Ilani . 2025. “Understanding the Determinants of Decision Readiness in Kidney Replacement Therapy: Shared Decision Making, Health Literacy, and Population‐Health Strategies.” Clinical Journal of the American Society of Nephrology 20, no. 7: 909–911. 40962369 10.2215/CJN.0000000762 PMC12262909

[nop270666-bib-0017] Li, Y. 2017. Construction and Application of Decision—Making Support Programs for Patients With Early‐Stage Primary Liver Cancer. Naval Medical University of the People's Liberation Army of China.

[nop270666-bib-0018] Liu, C. 2022. Construction and Application of a Risk Prediction and Assessment Index System for Disability in the Elderly Based on the Theory of Social Determinants of Health. North China University of Science and Technology.

[nop270666-bib-0019] Lu, S. L. 2022. Factors Influencing and Experiences of Cancer Patients' Participation in Treatment Decision‐Making. Shandong University.

[nop270666-bib-0020] Ma, L. L. 2004. Current Status of Cancer Patients' Participation in Treatment and Nursing Decision‐Making. Peking Union Medical College.

[nop270666-bib-0021] Mai, Q. , S. Xu , J. Hu , et al. 2023. “The Association Between Socioeconomic Status and Health‐Related Quality of Life Among Young and Middle‐Aged Maintenance Hemodialysis Patients: Multiple Mediation Modeling.” Frontiers in Psychiatry 14: 1234553. 37795510 10.3389/fpsyt.2023.1234553 PMC10546310

[nop270666-bib-0022] Noteboom, E. A. , A. M. May , E. van der Wall , N. J. de Wit , and C. W. Helsper . 2021. “Patients' Preferred and Perceived Level of Involvement in Decision Making for Cancer Treatment: A Systematic Review.” Psychooncology 30, no. 10: 1663–1679. 34146446 10.1002/pon.5750 PMC8518833

[nop270666-bib-0023] Pearce, K. E. , Y. Guo , S. Subhash , et al. 2025. “Decision Readiness and Determinants of KRT Among Veterans With Advanced CKD.” Clinical Journal of the American Society of Nephrology 20, no. 7: 931–939. 40215111 10.2215/CJN.0000000713 PMC12262920

[nop270666-bib-0024] Riley, R. D. , J. Ensor , K. I. E. Snell , et al. 2020. “Calculating the Sample Size Required for Developing a Clinical Prediction Model.” BMJ 368: m441. 32188600 10.1136/bmj.m441

[nop270666-bib-0025] Sabin, J. , E. Salas , J. Martín‐Martínez , et al. 2024. “Decisional Conflict Regarding Disease‐Modifying Treatment Choices Among Patients With Mid‐Stage Relapsing‐Remitting Multiple Sclerosis.” Patient Preference and Adherence 18: 1163–1171. 38863945 10.2147/PPA.S459242 PMC11166147

[nop270666-bib-0026] Song, X. P. , T. H. Xing , J. L. Ji , et al. 2025. “Current Status and Influencing Factors of Decision‐Making Readiness in HIV/AIDS Patients Undergoing Antiviral Therapy.” Dermatology and Venereology 47, no. 1: 26–29.

[nop270666-bib-0027] Stacey, D. , K. B. Lewis , M. Smith , et al. 2024. “Decision Aids for People Facing Health Treatment or Screening Decisions.” Cochrane Database of Systematic Reviews 1, no. 1: CD001431. 38284415 10.1002/14651858.CD001431.pub6 PMC10823577

[nop270666-bib-0028] Su, W. , J. Li , M. Zhai , et al. 2025. “Current Status and Influencing Factors of Decisional Conflict in Lung Cancer Patients Receiving Systemic Therapy: A Cross‐Sectional Analysis.” Thoracic Cancer 16, no. 16: e70150. 40817614 10.1111/1759-7714.70150 PMC12356992

[nop270666-bib-0029] Sui, Q. Y. 2021. Current Status and Influencing Factors of Decision‐Making Participation and Decision Regret in Breast Cancer Patients. Shandong University.

[nop270666-bib-0030] Sullivan, D. R. , J. P. Wisnivesky , S. M. Nugent , et al. 2023. “Decision Regret Among Patients With Early‐Stage Lung Cancer Undergoing Radiation Therapy or Surgical Resection.” Clinical Oncology (Royal College of Radiologists) 35, no. 6: e352–e361. 10.1016/j.clon.2023.03.015 PMC10241560 37031075

[nop270666-bib-0031] Sun, H. , H. Wang , N. Xu , et al. 2019. “Patient Preferences for Chemotherapy in the Treatment of Non‐Small Cell Lung Cancer: A Multicenter Discrete Choice Experiment Study in China.” Patient Preference and Adherence 13: 1701–1709. 31631985 10.2147/PPA.S224529 PMC6790116

[nop270666-bib-0032] Tan, N. Q. P. , S. P. E. Nishi , L. M. Lowenstein , et al. 2022. “Impact of the Shared Decision‐Making Process on Lung Cancer Screening Decisions.” Cancer Medicine 11, no. 3: 790–797. 34964284 10.1002/cam4.4445 PMC8817098

[nop270666-bib-0033] Tan, T. N. , J. Han , T. T. Fan , et al. 2023. “Latent Class Analysis of Decision‐Making Readiness in Young and Middle‐Aged Female Cancer Patients.” Military Nursing 40, no. 6: 25–29.

[nop270666-bib-0034] Wu, L. M. , S. S. Chiou , P. C. Lin , Y. M. Liao , and H. L. Su . 2022. “Decisional Conflicts, Anxiety, and Perceptions of Shared Decision‐Making in Cancer Treatment Trajectory Among Adolescents With Cancer: A Longitudinal Study.” Journal of Nursing Scholarship 54, no. 5: 589–597. 35238457 10.1111/jnu.12772

[nop270666-bib-0035] Zhang, P. P. , Y. M. Cheng , Z. R. Fang , et al. 2024. “Current Status and Influencing Factors of Decision—Making Readiness in Lung Cancer Patients Undergoing Chemotherapy.” Military Nursing 41, no. 6: 61–64.

