# Supplementary material for: Decision‐Making Readiness and Its Influencing Factors Among Lung Cancer Patients Receiving Chemotherapy: A Cross‐Sectional Study
Source: Nurs Open. 2026 Jun 29;13(7):e70666. doi: 10.1002/nop2.70666 (PMC13311732; doi:10.1002/nop2.70666)

**Table S1. Collinearity diagnostics of the DMA, DCS, DRS**

| **Coefficients^a^** | | | | | | | | |
| --- | --- | --- | --- | --- | --- | --- | --- | --- |
| Model | | Unstandardized Coefficients | | Standardized Coefficients | t | Sig. | Collinearity Statistics | |
|  |  | B | Std. Error | Beta |  |  | Tolerance | VIF |
| 1 | (Constant) | 24.300 | 2.458 |  | 9.886 | .000 |  |  |
|  | DMA | .420 | .069 | .269 | 6.113 | .000 | .836 | 1.197 |
|  | DCS | -.211 | .027 | -.380 | -7.950 | .000 | .710 | 1.409 |
|  | DRS | .186 | .102 | .080 | 1.821 | .069 | .831 | 1.203 |
| a. Dependent Variable: DMR | | | | | | | | |

**Figure S1. Scatter plot of unstandardized residuals versus unstandardized predicted values**


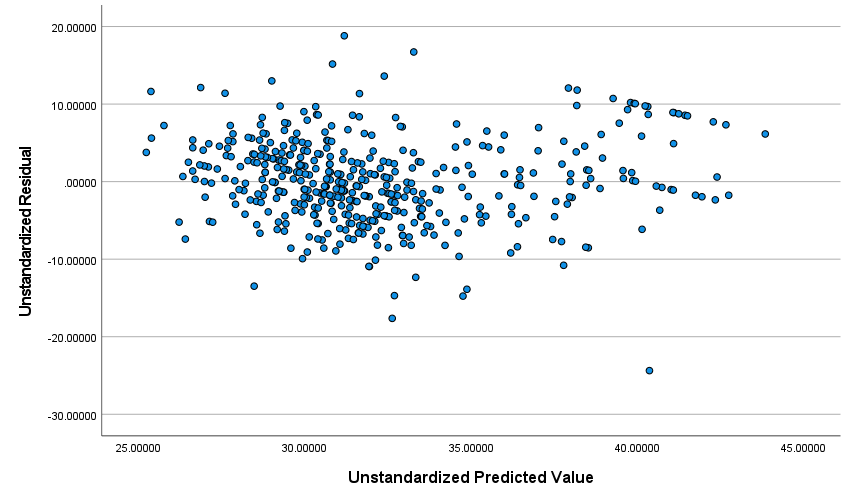


**Figure S2. Normal Q-Q Plot of Unstandardized Residuals for the Hierarchical Regression Model**


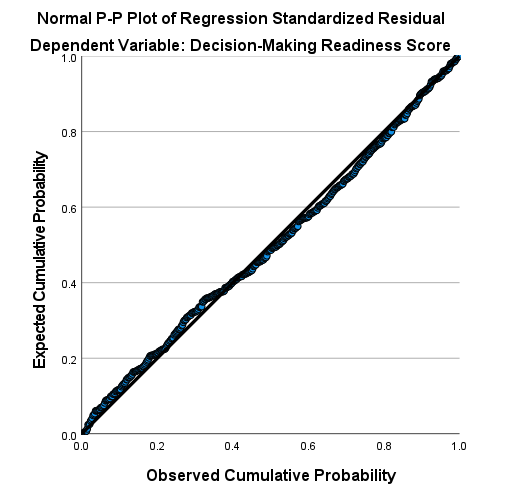

Supplement: Supplementary file 1 — Table S1: Collinearity diagnostics of the DMA, DCS and DRS. Figure S1: Scatter plot of unstandardized residuals versus unstandardized predicted values. Figure S2: Normal Q–Q Plot of unstandardized residuals for the Hierarchical Regression Model. [file NOP2-13-e70666-s001.docx]
