# Supplementary material for: Decision‐Making Readiness and Its Influencing Factors Among Lung Cancer Patients Receiving Chemotherapy: A Cross‐Sectional Study
Source: Nurs Open. 2026 Jun 29;13(7):e70666. doi: 10.1002/nop2.70666 (PMC13311732; doi:10.1002/nop2.70666)

**Table S2. Model Summary of the Hierarchical Regression Analysis**


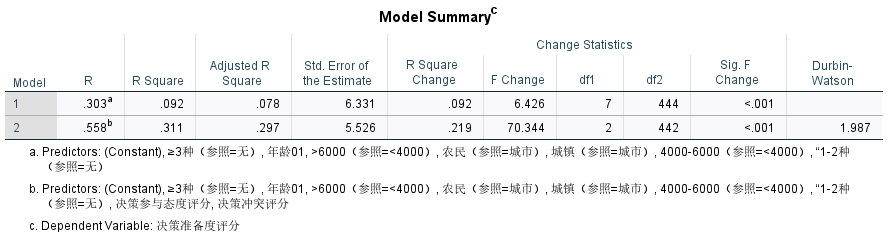


a. Predictors: (Constant), ≥3 types (reference = none), 1-2 types (reference = none), 18~59 years, >6000 RMB (reference = <4000RMB), country side (reference = urban), town (reference = urban), 4000-6000 (reference = <4000).

b. Predictors: (Constant), ≥3 types (reference = none), 18~59 years, >6000 RMB (reference = <4000 RMB), country side (reference = urban), town (reference = urban), 4000-6000 (reference = <4000), 1-2 types (reference = none), decision-making participation attitude score, decisional conflict score.

c. Dependent Variable: Decision-making readiness score.

**Figure S3. Normal Q-Q Plot of Unstandardized Residuals for the Hierarchical Regression Model**


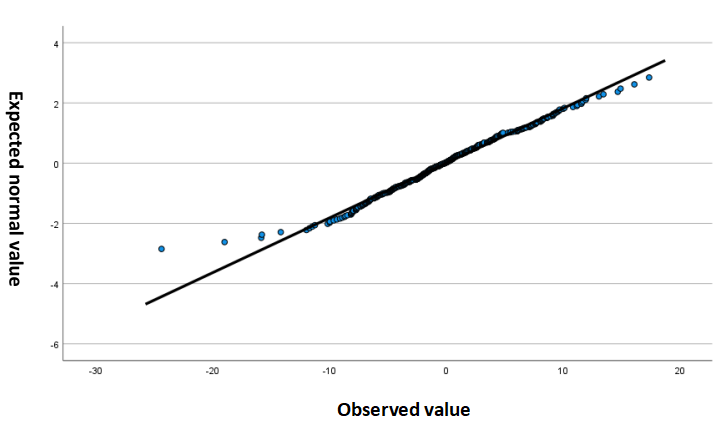


**Figure S4. Scatter Plot of Studentized Residual vs. Unstandardized Predicted Value**


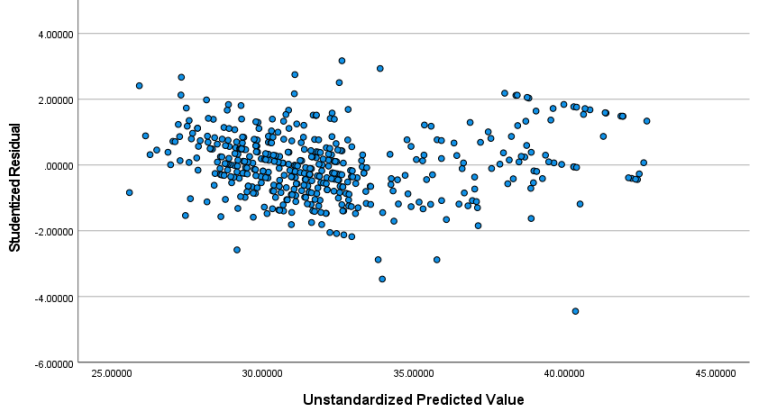


**Table S3. Coefficients table with collinearity statistics**


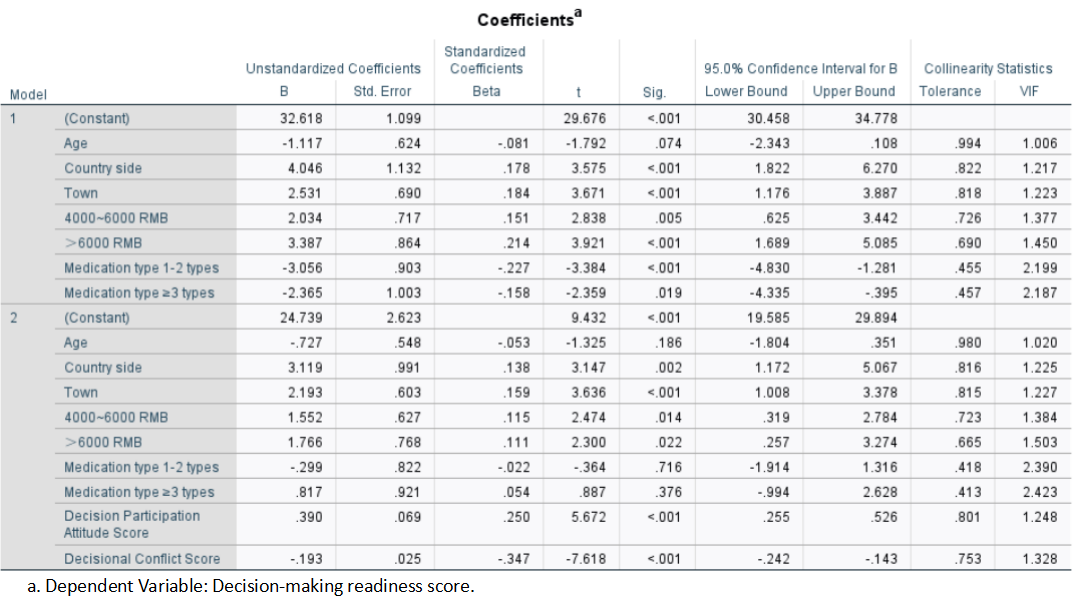

Supplement: Supplementary file 2 — Table S2: Model summary of the Hierarchical Regression Analysis. Figure S3: Normal Q–Q plot of unstandardized residuals for the Hierarchical Regression Model. Figure S4: Scatter plot of studentized residual versus unstandardized predicted value. Table S3: Coefficients table with collinearity statistics. [file NOP2-13-e70666-s002.docx]
